# Clinical and Microbial Etiology Characteristics in Pediatric Urinary Tract Infection

**DOI:** 10.3389/fped.2022.844797

**Published:** 2022-04-07

**Authors:** Jiandong Lu, Xiaozhu Liu, Yi Wei, Chengjun Yu, Jie Zhao, Ling Wang, Yang Hu, Guanghui Wei, Shengde Wu

**Affiliations:** ^1^Department of Urology, National Clinical Research Center for Child Health and Disorders, Ministry of Education Key Laboratory of Child Development and Disorders, Chongqing Key Laboratory of Pediatrics Chongqing, Children's Hospital of Chongqing Medical University, Chongqing, China; ^2^Department of Cardiology, The Second Affiliated Hospital of Chongqing Medical University, Chongqing, China

**Keywords:** urinary tract infection, urological abnormalities, congenital anomalies of the kidney and urinary tract (CAKUT), pediatric, children

## Abstract

**Background:**

Urinary tract infection (UTI) is a common occurrence in children. UTI and urological malformations are intimately linked. However, whether urinary tract malformations affect the clinical features of pediatric UTI remains unclear. The purpose of this study was to characterize the clinical features and microbial etiology of UTI in children.

**Methods:**

We retrospectively reviewed the records of 741 patients with UTI treated at the Chongqing Medical University Affiliated Children's Hospital between 2015 and 2020. Patients with and without urological malformations were compared using propensity score matching (PSM).

**Results:**

*Escherichia coli* was the most common causative microorganism of UTI, accounting for 40.5% of infections. One hundred twenty-two patients (16.5%) had urological malformations. PSM identified 122 matched pairs of patients with or without urological malformations. The proportion of patients with UTI caused by atypical microorganisms was significantly higher in patients with urological malformations (*P* = 0.048). Children with urological malformations showed longer duration of intravenous antibiotic treatment (*P* = 0.010), higher cost of treatment (*P* < 0.001), and higher prevalence of recurrence (23.8 vs. 10.7%, *P* < 0.001), compared with the normal group.

**Conclusion:**

Children with urological malformations are more likely to develop UTI with atypical microorganisms. Appropriate imaging examination and urine culture are strongly recommended for the diagnosis and management of pediatric UTI.

## Introduction

Urinary tract is the most common site of bacterial infection in the human body. Approximately 8% of individuals develop urinary tract infection (UTI) at some time during their childhood (1). The signs and symptoms of UTI in children are non-specific. Moreover, infants and young children cannot clearly articulate the symptoms. Thus, the diagnosis and treatment of UTI in children are commonly delayed, resulting in significant morbidity and sequelae including renal scarring and end-stage renal disease. UTI and its sequelae place a significant burden on the affected families and the society (2, 3).

Ascending infection is the predominant mode of occurrence of UTI. Microorganisms can pass into the urethra and ascend toward the bladder. *Escherichia coli* is the most frequently implicated microorganism in UTI with prevalence rates ranging from 50 to 60% (4). Both urinalysis and urine culture are required for diagnosis of UTI in children. However, urine culture is typically time consuming. Traditionally, empirical antimicrobial therapy is administered while awaiting the culture results. However, the indiscriminate use or misuse of antibiotics contributes to the emergence of multi-resistant bacteria. Indeed, in a recent study, Escherichia coli was identified in 75.7% of urine cultures and the overall positive rate of extended-spectrum β-lactamase (ESBL) in Enterobacterales was 37.2% (5). The emergence of antibiotic resistance is a global public health problem (6). It is also responsible for increasing treatment costs and drug adverse effects due to infections. However, whether this phenomenon has persisted over time remains unclear. Furthermore, appropriate antibiotic prescription requires knowledge of characteristics of UTI, the incidence of different pathogens, and local antibiogram of the pathogens. Taking this into account, one of the purposes of this article was to update the epidemiologic data of pediatric UTI in our region.

The rapid advances in imaging modalities over the last decades have enabled the detection of kidney and urinary tract diseases in individuals with UTI. Congenital anomalies of kidney and urinary tract are common in children. These anomalies are frequently associated with pediatric UTI (7–9). According to EAU/ESPU guidelines for UTIs, the presence of underlying urinary tract or kidney anatomical abnormalities is a risk factor for UTI (10). A systematic review and meta-analysis of 18 studies revealed that children with high grade of vesicoureteral reflux are prone to UTI and more likely to develop renal scars (11). However, the effect of urinary tract malformations on the bacterial composition is not well characterized. To this end, we performed a retrospective review and compared the clinical characteristics and microbiological etiology in 244 pediatric UTI patients with or without urinary tract malformations treated at our center. To the best of our knowledge, no similar investigation has ever been conducted on children in our region.

## Materials and Methods

This was a retrospective study conducted at the Chongqing Medical University Affiliated Children's Hospital. The study was approved by the institutional ethics committee.

We retrospectively reviewed electronic medical records of patients aged <18 years between January 2015 and December 2020. Only patients who had UTI that was confirmed by urine culture were included in the study. Data pertaining to clinical characteristics, socio-demographic characteristics, imaging evaluation, laboratory findings, and risk factors associated with UTI were collected. Data pertaining to the cost of treatment were obtained from the hospital financial database. Patients with incomplete records were excluded. Our patients were all in-hospital patients, and both upper and lower UTIs were included. For patients with multiple hospital admissions, only the last admission record was used. Patients who were undergoing antibiotic prophylaxis, antibiotic treatment within 48 h before hospital admission, and catheterization within the previous 2 weeks were excluded.

Clean catch void, suprapubic aspiration, urethral catheterization, and urine collection bag were used to obtain urine specimens. Consistent with American Academy of Pediatrics guidelines (12), bladder catheterization was the main method used in our cohort.

UTI was defined as a positive culture with urinary symptoms.

Positive test result of urine culture was defined as a single pathogen growth of more than 10^5^ colony-forming units/mL (CFU/mL) from a clean catch specimen; or at least 50,000 CFU/mL of a single uropathogen from a catheterized specimen; or any uropathogenic bacteria from a suprapubic aspirated urine.

Patients were categorized into two groups according to the presence or absence of anomalies of the kidney and urinary tract. The common underlying renal and urinary tract anomalies in children are hydronephrosis, vesicoureteral reflux, ureter ectasis, urolithiasis, ureterocele, renal dysplasia or hypoplasia, renal duplication, ureteral duplication, posterior urethral valve, single kidney, and vesical diverticula. Patients with the above anatomic abnormalities were included in the anomaly group. The remaining patients were included in non-anomaly group. Pyuria was defined as ≥10 white blood cells per high power field on urine microscopy.

Subsequently, we selected age, sex, and circumcision in male patients for propensity score generation. To minimize selection bias, 1:1 propensity score–matching (PSM) analysis was performed.

### Statistical Analysis

SPSS Statistics 22 (IMB, Armonk, NY) was used for data processing and analysis. Continuous variables are expressed as mean ± standard deviation or median ± interquartile range (IQR), and categorical variables are expressed as frequency (%). We used descriptive statistics to summarize socio-demographic data and pathogen features. Between-group differences were assessed using the Mann-Whitney U test or Chi-squared tests. *P*-values < 0.05 were considered indicative of statistical significance. PSM analysis was performed with IBM SPSS Statistics version 22 (IMB, Armonk, NY) and R software (Version 3.3.2). The 1:1 matched analysis was performed using nearest-neighbor matching without replacement. A caliper distance of 0.2 was selected.

## Results

### Characteristics of the Study Population

A total of 741 children diagnosed with UTI were identified. The demographic characteristics are presented in Table 1. Of these, 589 (78.9%) of patients had a first episode of UTI and 156 (21.1%) had recurrent UTI. The median age at presentation was 2.0 years (IQR = 6.0). Approximately 60% of patients had disease onset at the age of <4 years. Males were predominant (51.1%) and only 43 (11.3%) of them were circumcised. The age stratified results presented in Table 2 suggest that UTI affects male and female children approximately equally across age groups (*P* = 0.785).

**Table 1:** Baseline characteristics of the study population.

	**Patients included (*n* = 741)**
**Male**, ***n*** **(%)**	379 (51.1)
**Female**, ***n*** **(%)**	362 (48.9)
**Age (years), median (IQR)**	2 (1–7)
**Circumcised**, ***n*** **(%)**	
Yes	43 (5.8)
No	686 (92.6)
Unknown	12 (1.6)
**History of urinary tract infection**, ***n*** **(%)**	
<2	585 (78.9)
≥2	156 (21.1)
**Fever**, ***n*** **(%)**	89 (12.1)
**Antibiotic treatment in the previous 2 weeks**, ***n*** **(%)**	76 (10.2)
**Urological malformations**, ***n*** **(%)**	122 (16.5)
**Type of strain isolated**, ***n*** **(%)**	
Gram-negative	523 (70.6)
Gram-positive	218 (29.4)

*IQR, inter-quartile range*.

**Table 2:** Age and sex distribution of 741 patients with UTI.

**Age (years)**	**Sex**		**Total**	**%**
	**Males**	**Females**		
<1	60	61	121	16.3
1–3	161	159	320	43.2
4–18	158	142	300	40.5
Total	379 (51.1)	362 (48.9)	741	100

Methods of urine collection for culture were urethral catheterization (77.8%), clean catch void (12.5%), urine collection bag (7.4%) and suprapubic aspiration (2.3%).

### The Microbial Spectrum of UTI Based on Urinary Culture

**Figure 1:**
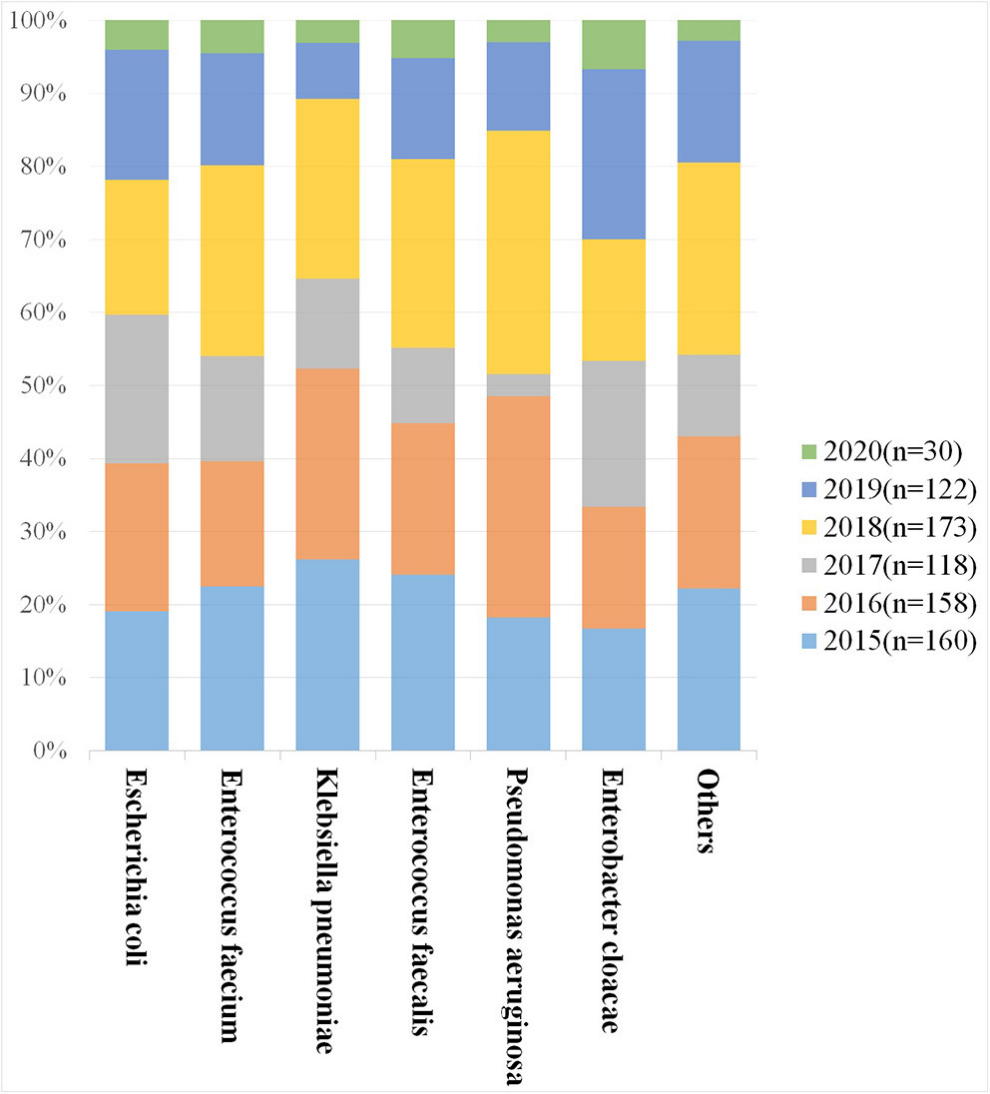
The distribution of bacterium isolated over six years.

Gram-negative organisms accounted for 70.6% of all cases of UTI. The most common isolated pathogen was *Escherichia coli*, which was found in 40.5% (300/741) of patients, followed by *Enterococcus faecium* in 15.0% (111/741) of patients, *Klebsiella* species in 8.9% (66/741) of patients, *Enterococcus faecalis* in 7.8% (58/741) of patients, *Pseudomonas aeruginosa* in 4.3% (32/741) of patients, and *Enterobacter cloacae* in 4.1% (30/741) of patients (Table 3). The distribution of bacterium isolated in urine during the study period is depicted in Figure 1.

**Table 3:** Etiological agents in children with urinary tract infection.

**Etiological agents**	***n***	**%**
Escherichia coli	300	40.5
Enterococcus faecium	111	15.0
Klebsiella pneumoniae	66	8.9
Enterococcus faecalis	58	7.8
Pseudomonas aeruginosa	32	4.3
Enterobacter cloacae	30	4.1
Morganella fulton	16	2.2
Staphylococcus epidermidis	15	2.0
Klebsiella oxytoca	12	1.6
Acinetobacter baumannii	10	1.4
Staphylococcus hemolyticus	9	1.2
Enterobacter aerogenes	7	0.9
Serratia marcescens	6	0.8
Staphylococcus aureus	5	0.7
C. albicans	5	0.7
Enterococcus raffinosus	4	0.5
Citrobacter freundii	4	0.5
Others	51	6.9
Total	741	100

### Anomalies of the Kidney and Urinary Tract in UTI

In our cohort, 16.5% of patients had ≥1 defect in the kidney or urinary tract. Hydronephrosis was the most common malformation accounting for 42.3% of patients, followed by vesicoureteral reflux (17.0%) and ureterectasis (12.4%). Accordingly, 122 patients who had urological malformations were included in the anomaly group. There was no difference in sex distribution between the anomaly and non-anomaly groups (*P* = 0.132). However, there were significant between-group differences with respect to age, history of circumcision, and history of previous chemotherapy or chemoradiation (*P* < 0.001, *P* = 0.021, and *P* < 0.001, respectively).

### Characteristics of Patients After Propensity Score Matching

After PSM, 112 matched pairs of patients were identified from both groups. Table 4 presents the characteristics of the propensity score matched cohort. There were no significant between-group differences with respect to demographic characteristics.

**Table 4:** Characteristics of patients in the propensity score matched cohort.

	**Anomaly group** **(*n* = 122)**	**Non-anomaly group** **(*n* = 122)**	***P*-value**
**Age (years), median (IQR)**	1.0 (0.8–4.0)	2.0 (1.0–6.0)	0.111
**Age (years), mean (SD)**	3.1 (4.0)	3.7 (3.8)	0.178
**Sex ratio (male:female)**	70:52	61:61	0.262
**Symptoms at onset**			0.045
Poor feeding	19 (15.6)	26 (21.3)	0.248
Vomiting	32 (26.2)	19 (15.6)	0.041
Failure to thrive	29 (22.1)	31 (25.4)	0.766
Strong-smelling urine	9 (7.3)	11 (9.0)	0.641
Irritability	25 (20.5)	21 (17.2)	0.76
Fever	18 (14.7)	11 (9.0)	0.018
Gastrointestinal complaints	8 (6.6)	16 (13.1)	0.085
Abdominal or flank pain	31 (25.4)	27 (22.1)	0.547
Dysuria	19 (15.6)	16 (13.1)	0.584
Urgency	15 (12.3)	22 (18.0)	0.212
Urinary frequency	15 (12.3)	22 (18.0)	0.212
Other specific symptoms	41 (33.6)	34 (27.9)	0.331
**Laboratory measurements**			
Pyuria, *n* (%)	92 (75.4)	99(81.1)	0.337
Urine nitrite positive, *n* (%)	20 (16.4)	13 (10.7)	0.017
C-reactive protein (mg/L), median (SD)	21.8 (31.5)	13.8 (22.2)	0.012
Neutrophilic granulocyte percentage (%), median (IQR)	50.7 (40.0–62.0)	50.4 (37.8–64.2)	0.918
Serum creatinine (μmol/L), median (IQR)	27.0 (22.0–39.3)	26.0 (21.0–40.0)	0.758
**Type of strain isolated**, ***n*** **(%)**			0.887
Gram–	87 (71.3)	88 (72.1)	
Gram+	35 (28.7)	34 (27.9)	
**Organism**, ***n*** **(%)**			0.042
*Escherichia coli*	52 (42.6)	48 (39.3)	0.603
*Enterococcus faecium*	22 (18.0)	18 (14.8)	0.489
*Klebsiella pneumoniae*	15 (12.3)	12 (9.8)	0.504
*Enterococcus faecalis*	8 (6.6)	6 (4.9)	0.583
*Pseudomonas aeruginosa*	5 (4.1)	7 (5.7)	0.554
*Enterobacter cloacae*	4 (3.3)	5 (4.1)	0.734
*Klebsiella oxytoca*	3 (2.5)	2 (1.6)	0.651
Others	13 (10.6)	24 (19.8)	0.048
**Hospitalization costs (CNY), median (IQR)**	11,834.5 (8,117.0–20,292.7)	8,586.6 (6,423.1–15,269.8)	0.010
**Duration of antibiotic treatment (days), median (IQR)**	13.0 (14.0–21.0)	9.0 (7.0–14.0)	<0.001
**>1 episode of UTI**, ***n*** **(%)**	29 (23.8)	13 (10.7)	0.009

*IQR, inter-quartile range; SD, standard deviation; CNY, China Yuan*.

### Clinical Outcomes After PSM

Symptoms at onset of UTI were non-specific and vague. In both groups, the chief complaints were poor feeding, vomiting, failure to thrive, strong-smelling urine, irritability, fever, gastrointestinal complaints, abdominal or flank pain, dysuria, urgency, and/or urinary frequency. There was a significant difference with respect to symptom distribution between the anomaly and non-anomaly groups (*P* = 0.045). In the anomaly group, vomiting, abdominal or flank pain, and failure to thrive were the most common symptoms at presentation (26.2, 25.4, and 22.1%, respectively). The prevalence of vomiting was significantly more common in the anomaly group than in the non-anomaly group (26.2 vs. 15.6%; *P* = 0.041).

Pyuria is a comparatively frequent laboratory finding. The prevalence of pyuria in patients with urinary tract abnormalities was not significantly different from that of the normal group (75.4 vs. 81.1%; *P* = 0.337). In addition, a urine nitrite test was positive in 20 patients in the anomaly group and 13 patients in the non-anomaly group; the between-group difference in this respect was statistically significant (*P* = 0.017). C-reactive protein level in the anomaly group was significantly higher than that in the non-anomaly group (*P* = 0.012). There were no significant between-group differences with respect to percentage of neutrophilic granulocytes, white blood cell count, or serum creatinine.

Gram-negative microorganisms were isolated in 71.3% patients in the anomaly group and 72.1% patients in the non-anomaly group (*P* = 0.887). The types of causative organism were significantly different in the anomaly group and non-anomaly group (*P* = 0.042). In the anomaly group, the most common causative organisms were *Escherichia coli, Enterococcus faecium, Klebsiella pneumoniae, and Enterococcus faecalis*, accounting for 42.6, 18.0, 12.3, and 6.6% of pathogens, respectively. No significant difference was observed in the prevalence of 7 most common causative organisms between the two groups. However, other organisms which are relatively rare in UTI were significantly different between the groups (*P* = 0.048).

In our cohort, the most commonly prescribed class of antibiotics for UTI was piperacillin/tazobactam (407 cases, 54.9%), followed by latamoxef (179 cases, 22.4%). In the anomaly group, 25.4% patients were switched to narrower spectrum agents from previous antibiotics upon identification of causative microorganisms by urine culture. There was no significant between-group difference with respect to the rate of change in antibiotic (25.4 vs. 18.8%; *P* = 0.217). Urine culture conversion to negative had occurred in 51.4% patients during the hospital stay. The median time to conversion to a negative urine culture was 5.6 ± 4.3 days.

Costs were determined from billing data. It comprised primarily drug cost, diagnostic cost and routine services cost. Drug costs were identified as a major source of cost burden which were mainly affected by the length of stay. The analysis revealed a significant increase in treatment costs in the anomaly group compared with those in the non-anomaly group (*P* < 0.001). Moreover, the anomaly group showed a longer duration of intravenous antibiotic treatment during inpatient treatment compared with the non-anomaly group (*P* = 0.010) and higher prevalence of recurrence (23.8 vs. 10.7%, *P* < 0.001).

## Discussion

Over the 6-year reference period for this study, *E*. *coli* was the most common pathogen isolated in urine samples from patients with UTI treated at our hospital. This finding is consistent with previous studies (9, 13, 14).

The median age of patients in our cohort was 2 years, which is lower than the median age of >3 years reported by Weisz et al. (15). Male children accounted for a slightly higher proportion of study population than female children. This finding is contrary to previous studies in which female preponderance was found in UTI (16–18). A possible explanation for this might be that male children are more affected by urological malformations than female children. Anatomically, urethral valve and phimosis in male children can result in obstruction, increasing the risk of UTI. Compared with men, women have a shorter urethra in close proximity to genital organs, which provides microorganisms with easy access to the urinary tract. In spite of this, the factor cannot be overestimated due to the fact that female children of younger age did not experience menstruation and sexual activity. Another possible explanation could be related to circumcision. In our cohort, only 11.3% of male children were circumcised. This proportion was substantially less than that reported elsewhere. Circumcision was more prevalent in the United States and the reported circumcision rate in neonates was up to 75% (19). In non-circumcised male children, bacterial flora may adhere well to foreskin mucosa increasing the risk of UTI. A meta-analysis by Buscemi et al. (20) suggested a lifetime benefit from circumcision due to reduced risk of UTI. The American Academy of Pediatrics also advocates the benefits of male circumcision with respect to prevention of UTI (21). Thus, physicians should explain the potential benefits and risks of circumcision to parents of children who are at high risk of UTI, to ensure they have sufficient knowledge to permit informed decision-making.

We investigated the clinical, radiological, and etiological characteristics of UTI in children with or without malformations of the urinary tract. In addition, we performed PSM analysis to minimize the influence of confounding variables. To the best of our knowledge, this is the first study to compare the clinical outcomes in different groups in UTI utilizing PSM. In our study, pediatric patients with UTI with or without urinary tract malformations had similar clinical symptoms and laboratory measurements. This is consistent with previous studies which showed that the clinical and laboratory features of UTI in children are non-specific (8, 9, 22, 23). This often contributes to the delayed diagnosis and management of UTI in pediatric patients.

At present, a positive urine culture result is considered as the gold standard for diagnosis of UTI (24). In our cohort, *E. coli* was the predominant causative microorganism in pediatric UTI, regardless of the group. We also found that identification of atypical species in children with urological malformations was almost double compared to that in the non-anomaly group. Previous studies have identified a potential association between non-*E. coli* UTI and urological malformations (25–27). In a study conducted in Cuba, the presence of VUR was a significant risk factor for non-*E. coli* UTI (26). Pauchard et al. (27) also showed UTI caused by non-*E. coli* is frequently associated with the presence of an anatomical malformation. This is consistent with our findings. Unlike these studies, we further explored the distribution of bacterial species other than *E. coli* in detail. We cautiously speculate that children with urological malformations are more likely to develop UTI caused by atypical microorganisms, such as *Staphylococcus epidermidis*. This interesting result may be related to alteration in the bacterial composition of urinary tract. A recent review article mentioned that the urinary tract is not sterile and an imbalance in urinary microbiome may contribute to the development of UTI (28). Normally, bacteria in the urinary tract are washed away by the unidirectional flow of urine in healthy individuals. Malformations of the urinary tract or kidney may slow down the urine flow and even lead to urinary reflux, which may increase the odds of infection caused by atypical microorganisms. However, these hypotheses need to be further investigated and validated.

The number of patients included in this study was much lower than our expectation. This may have been attributable to several factors. First, one of the key reasons was the dramatic decline in medical admissions due to the impact of COVID-19 (29). Indeed, the number of patients included in 2020 was 75.4% lower than that in 2019 in our study. Typically, UTI in pediatric patients is considered as a less urgent condition. Due to concerns related to the epidemic, parents may have avoided taking their children to the hospital. However, the consequent delay in seeking treatment may have serious consequences in children with UTI. Consequently, better public health policies are required to ensure that patients with similar situations can obtain hospital care during the pandemic. The second reason could have been because of insufficient attention to urine culture. This may be due to negligence of both the medical staff and the patient. During data collection, we noted that a significant proportion of patients who were clinically diagnosed with UTI did not undergo urine culture test at least once. When compared with other physicians, pediatricians are more likely to obtain urine cultures (30). In addition, our study population has a relatively young age. Suprapubic aspiration and urethral catheterization are recommended in the non–toilet-trained or initial trained population for urine collection (31). However, both of methods are invasive. Parents often do not consent to this procedure because it can cause discomfort to their children. This reminds us that obtaining a proper urine culture requires engagement with medical staff and education of families.

Some limitations of our study should be acknowledged. First, due to the retrospective study design, the influence of selection bias on our results cannot be ruled out. Second, the sample size was relatively small. The rigorous inclusion criteria restricted the sample size. A large number of patients were excluded because of lack of urine culture results or negative urine culture results. Medical admissions fell dramatically under the influence of COVID-19 outbreak, as mentioned before. Third, the study population was largely sourced from a single area in China and we did not include outpatient data in the analysis for comparison. Fourth, the proportion of lower and upper UTIs were not specified. Based on the retrospective data, we were unable to accurately differentiate children with cystitis from children with pyelonephritis. Finally, due to the insufficient data, this study does not discuss the degree of hydronephrosis and urinary tract infection was not classified by infection site. Our findings may not be generalizable to other populations. Hence, our findings need to be interpreted cautiously. Further, multicenter studies with rigorous design should be performed in the future.

## Conclusion

In conclusion, *E. coli* was the predominant causative microorganism of pediatric UTI in our study. Children with urological malformations are more likely to develop UTI caused by atypical microorganisms, which could lead to longer hospital stay, high health care costs, and high recurrence. Characterizing the clinical features and microbial etiology can facilitate the timely diagnosis and treatment of pediatric UTI.

## Data Availability Statement

The raw data supporting the conclusions of this article will be made available by the authors, without undue reservation.

## Ethics Statement

The studies involving human participants were reviewed and approved by the Institutional Ethics Committee of Chongqing Medical University Affiliated Children's Hospital. Written informed consent from the participants' legal guardian/next of kin was not required to participate in this study in accordance with the national legislation and the institutional requirements.

## Author Contributions

GW, SW, and XL participated in the design of this study. JL, YW, CY, and JZ performed the statistical analysis. XL, LW, YH, and JL carried out the study and collected important background information. JL drafted the manuscript. All authors read and approved the final manuscript.

## Funding

This work was supported by the National Natural Science Foundation of China (No. 81873828).

## Conflict of Interest

The authors declare that the research was conducted in the absence of any commercial or financial relationships that could be construed as a potential conflict of interest.

## Publisher's Note

All claims expressed in this article are solely those of the authors and do not necessarily represent those of their affiliated organizations, or those of the publisher, the editors and the reviewers. Any product that may be evaluated in this article, or claim that may be made by its manufacturer, is not guaranteed or endorsed by the publisher.

## References

[1] Mårild S Jodal U. Incidence rate of first-time symptomatic urinary tract infection in children under 6 years of age. Acta Paediatr. (1998) 87:549–52. 10.1111/j.1651-2227.1998.tb01502.x 9641738

[2] MacVane SH Tuttle LO Nicolau DP. Demography and burden of care associated with patients readmitted for urinary tract infection. J Microbiol Immunol Infect. (2015) 48:517–24. 10.1016/j.jmii.2014.04.002 24863498

[3] Steiger SN Comito RR Nicolau DP. Clinical and economic implications of urinary tract infections. Expert Rev Pharmacoecon Outcomes Res. (2017) 17:377–83. 10.1080/14737167.2017.1358618 28730918

[4] Sorlozano A Jimenez-Pacheco A de Dios Luna Del Castillo J. Evolution of the resistance to antibiotics of bacteria involved in urinary tract infections: a 7-year surveillance study. Am J Infect Control. (2014) 42:1033–8. 10.1016/j.ajic.2014.06.013 25278389

[5] Quan J Dai H Liao W Zhao D Shi Q Zhang L. Etiology and prevalence of ESBLs in adult community-onset urinary tract infections in East China: a prospective multicenter study. J Infect. (2021) 83:175–81 10.1016/j.jinf.2021.06.004 34116075

[6] Progress on antibiotic resistance. Nature. (2018) 562:307. 10.1038/d41586-018-07031-7 30333595

[7] Jodal U. The natural history of bacteriuria in childhood. Infect Dis Clin North Am. (1987) 1:713–29. 10.1016/S0891-5520(20)30146-X 3333655

[8] Arshad M Seed PC. Urinary tract infections in the infant. Clin Perinatol. (2015) 42:17–28, vii. 10.1016/j.clp.2014.10.003 25677994 PMC5511626

[9] Tullus K Shaikh N. Urinary tract infections in children. Lancet. (2020) 395:1659–68. 10.1016/S0140-6736(20)30676-0 32446408 PMC13498406

[10] Stein R Dogan HS Hoebeke P Kočvara R Nijman RJ Radmayr C. Urinary tract infections in children: EAU/ESPU guidelines. Eur Urol. (2015) 67:546–58. 10.1016/j.eururo.2014.11.007 25477258

[11] Najafi F Sarokhani D Hasanpour Dehkordi A. The prevalence of kidney scarring due to urinary tract infection in Iranian children: a systematic review and meta-analysis. J Pediatr Urol. (2019) 15:300–8. 10.1016/j.jpurol.2019.05.011 31229416

[12] Roberts KB. Urinary tract infection: clinical practice guideline for the diagnosis and management of the initial UTI in febrile infants and children 2 to 24 months. Pediatrics. (2011) 128:595–610. 10.1542/peds.2011-1330 21873693

[13] Lemberger U Quhal F Bruchbacher A Shariat SF Hiess M. The microbiome in urinary tract infections in children – an update. Curr Opin Urol. (2021) 31:147–54. 10.1097/MOU.0000000000000858 33449574

[14] Okarska-Napiera?A M Wasilewska A Kuchar E. Urinary tract infection in children: diagnosis, treatment, imaging - comparison of current guidelines. J Pediatr Urol. (2017) 13:567–73. 10.1016/j.jpurol.2017.07.018 28986090

[15] Amornchaicharoensuk Y. Clinical characteristics and antibiotic resistance pattern of pathogens in pediatric urinary tract infection. Southeast Asian J Trop Med Public Health. (2016) 47:976–82. 29620804

[16] Winberg J Andersen HJ Bergstr?M T Jacobsson B Larson H Lincoln K. Epidemiology of symptomatic urinary tract infection in childhood. Acta Pdiatrica. (2010) 63:1–20. 10.1111/j.1651-2227.1974.tb05718.x 4618418

[17] Hanna-Wakim RH Ghanem ST El Helou MW. Epidemiology and characteristics of urinary tract infections in children and adolescents. Front Cell Infect Microbiol. (2015) 5:45. 10.3389/fcimb.2015.00045 26075187 PMC4443253

[18] Ganesh R Shrestha D Bhattachan B Rai G. Epidemiology of urinary tract infection and antimicrobial resistance in a pediatric hospital in Nepal. BMC Infect Dis. (2019) 19:420. 10.1186/s12879-019-3997-0 31088380 PMC6518643

[19] Eisenberg ML Galusha D Kennedy WA Cullen MR. The relationship between neonatal circumcision, urinary tract infection, and health. World J Mens Health. (2018) 36:176–82. 10.5534/wjmh.180006 29623700 PMC6119846

[20] Morris BJ Wiswell TE. Circumcision and lifetime risk of urinary tract infection: a systematic review and meta-analysis. J Urol. (2013) 189:2118–24. 10.1016/j.juro.2012.11.114 23201382

[21] Male circumcision. Pediatrics. (2012) 130:e756–85. 10.1542/peds.2012-1990 22926175

[22] Brkic S Mustafic S Nuhbegovic S Ljuca F Gavran L. Clinical and epidemiology characteristics of urinary tract infections in childhood. Med Arh. (2010) 64:135–8. 20645503

[23] Lo DS Rodrigues L Koch V Gilio AE. Clinical and laboratory features of urinary tract infections in young infants. J Bras Nefrol. (2018) 40:66–72. 10.1590/1678-4685-jbn-3602 29796576 PMC6533974

[24] Hudson A Romao R MacLellan D. Urinary tract infection in children. CMAJ. (2017) 189:E608. 10.1503/cmaj.160656 28438954 PMC5403644

[25] Honkinen O Lehtonen OP Ruuskanen O Huovinen P Mertsola J. Cohort study of bacterial species causing urinary tract infection and urinary tract abnormalities in children. BMJ. (1999) 318:770–1. 10.1136/bmj.318.7186.770 10082700 PMC27791

[26] Díaz Álvarez M Acosta Batista B Pérez Córdova R Hernández Robledo E. Urinary tract infection caused by Enterobacteriaceae and its relationship with vesicoureteral reflux. Boletin medico del Hospital Infantil de Mexico. (2017) 74:34–40. 10.1016/j.bmhime.2016.10.001 29364812

[27] Pauchard JY Chehade H Kies CZ Girardin E Cachat F Gehri M. Avoidance of voiding cystourethrography in infants younger than 3 months with *Escherichia coli* urinary tract infection and normal renal ultrasound. Arch Dis Child. (2017) 102:804–8. 10.1136/archdischild-2016-311587 28408468

[28] Hiergeist A Gessner A. Clinical implications of the microbiome in urinary tract diseases. Curr Opin Urol. (2017) 27:93–8. 10.1097/MOU.0000000000000367 27898455

[29] Birkmeyer JD Barnato A Birkmeyer N Bessler R Skinner J. The impact of the COVID-19 pandemic on hospital admissions in the United States. Health Aff. (2020) 39:2010–7. 10.1377/hlthaff.2020.00980 32970495 PMC7769002

[30] Copp HL Yiee JH Smith A Hanley J Saigal CS. Use of urine testing in outpatients treated for urinary tract infection. Pediatrics. (2013) 132:437–44. 10.1542/peds.2012-3135 23918886 PMC3876750

[31] May OW. Urine collection methods in children: which is the best. Nurs Clin North Am. (2018) 53:137–43. 10.1016/j.cnur.2018.01.001 29779508

